# 

**Published:** 1897-09

**Authors:** 


### CONTENTS.

ORIGINAL COMMUNICATIONS—
Tropical Abscess of the Liver. By Edward B. Jackson, M.D., Houston, Texas.. 433
Threatened Death During Major Anesthesia. By Robert H. M. Dawbarn, M.D., New York.... 439
Two Cases of Concussion of the Brain. By M. A. Clark, A.M., M.D., Macon, Ga. 448
Trigeminal Paresthesia. By Curran Pope, M.D., Louisville, Ky.... 452
SOCIETY REPORTS—
Atlanta Society of Medicine.... 458
CORRESPONDENCE—
New York Letter.... 465
Notes from London.... 470
Study of the American Medicinal Flora.... 473
EDITORIAL—
Mob Law and Its Remedy.... 477
SELECTIONS AND ABSTRACTS—
Skin Diseases and Syphilis.... 481
Miscellaneous.... 486
MEDICAL ITEMS.... 496
BOOK REVIEWS.... 499
MEDICAL ITEMS—Continued.... x, xii, xiv, xvii
ADVERTISERS' INDEX TO ADVERTISEMENTS.... xviii
CLASSIFIED INDEX TO ADVERTISEMENTS.... xxxix

